# HP-LSP: A reference of land surface phenology from fused Harmonized Landsat and Sentinel-2 with PhenoCam data

**DOI:** 10.1038/s41597-023-02605-1

**Published:** 2023-10-11

**Authors:** Khuong H. Tran, Xiaoyang Zhang, Yongchang Ye, Yu Shen, Shuai Gao, Yuxia Liu, Andrew Richardson

**Affiliations:** 1 Geospatial Sciences Center of Excellence, Department of Geography & Geospatial Sciences, South Dakota State University, Brookings, SD 57007 USA; 2 School of Informatics, Computing, and Cyber Security, Northern Arizona University, Flagstaff, AZ 86011 USA; 3 Center for Ecosystem Science and Society, Northern Arizona University, Flagstaff, AZ 86011 USA

**Keywords:** Environmental sciences, Ecosystem ecology

## Abstract

Land surface phenology (LSP) products are currently of large uncertainties due to cloud contaminations and other impacts in temporal satellite observations and they have been poorly validated because of the lack of spatially comparable ground measurements. This study provided a reference dataset of gap-free time series and phenological dates by fusing the Harmonized Landsat 8 and Sentinel-2 (HLS) observations with near-surface PhenoCam time series for 78 regions of 10 × 10 km^2^ across ecosystems in North America during 2019 and 2020. The HLS-PhenoCam LSP (HP-LSP) reference dataset at 30 m pixels is composed of: (1) 3-day synthetic gap-free EVI2 (two-band Enhanced Vegetation Index) time series that are physically meaningful to monitor the vegetation development across heterogeneous levels, train models (e.g., machine learning) for land surface mapping, and extract phenometrics from various methods; and (2) four key phenological dates (accuracy ≤5 days) that are spatially continuous and scalable, which are applicable to validate various satellite-based phenology products (e.g., global MODIS/VIIRS LSP), develop phenological models, and analyze climate impacts on terrestrial ecosystems.

## Background & Summary

Phenological dynamic of terrestrial ecosystems is one of the most sensitive indicators of environmental and climate changes because it regulates water, energy, and carbon fluxes between the land and atmosphere^1–5^. In contrast to traditional methods for observing specific plant species through ground-based measurements and visual observations in small areas, land surface phenology (LSP) has been generated across regional to global scales by analyzing time series of vegetation indices observed from satellite observation systems^6–13^. In particular, the long-term global LSP at 500 m spatial resolution has been generated using the daily Moderate Resolution Imaging Spectroradiometer (MODIS) and Visible Infrared Imaging Radiometer Suite (VIIRS) observations since 2000^14,15^. Moreover, LSP at relatively homogenous vegetation communities across local and continental scales has been increasingly detected from satellite observations at 10–30 m spatial resolutions (e.g., 10–20 m Sentinel-2A/2B with 5-day revisit frequency and 30 m Harmonized Landsat 8/9 and Sentinel-2A/2B (HLS) with a temporal resolution of 2-3 days)^10,16–19^. More recently, LSP has been investigated using the daily commercial PlanetScope imagery at 3 m pixels acquired by a constellation of 200+ CubeSats^20,21^.

Although many LSP datasets have been increasingly produced at different spatial resolutions, the low quality of temporal satellite-based time series caused by various factors (including instrument-related errors, atmosphere, missing observations, and snow/cloud covers) is the most critical challenge leading to large uncertainties in the LSP detections^22,23^. The cloud cover could during a year consecutively last for a 16-day period in as much as 36% of Earth’s land surface in 2000 and for two months in 16% areas in 2001^9,24^, causing large gaps in the optical satellite time series. Although cloud-caused gaps are more frequent in tropical and subtropical climates, long-term gaps also occur during the winter period and spring rainy season in the temperate climate^23^. Thus, the use of quality assurance (QA) flags (usually provided in the satellite products) to remove low-quality observations (snow/ice, cloud, cloud shadow, adjacent cloud, and cirrus clouds) is an essential step in detecting phenological transition dates^10,23,25^. However, the abnormal values still likely appear in the satellite-derived time series after applying the QA flags because of the inaccurate atmospheric correction, residual cloud and snow, and other factors^15,26,27^. Such gaps and contaminations have very strong impacts on the accuracy of phenology detections. The discrepancy of more than 20 days in satellite-retrieved phenological timings has been noticed in the frequently-clouded regions^22^ and the uncertainty could be much larger if the gaps happen around the phenological transition dates^28^.

Various statistical methods have been developed to smooth the satellite time series by removing noise and filling gaps. The commonly used methods include maximum value composite (MVC)^29^, Savitzky-Golay filter^30^, moving average^31^, composite of multiple-year observations^32^, Fourier fitting^33,34^, polynomial curve fitting^35^, spline filter^14^, non-linear harmonic model based on a sequence of sines and cosines over time^36^, and integration of multiple approaches (moving average, moving median, Savitzky-Golay, and background determinations)^25^. Nevertheless, the smoothed time series is often under- or over-fitted if long-term gaps arise^36,37^. In other words, simply applying the smoothing approaches always fails to reconstruct the actual seasonal dynamics and detect accurate phenological timing due to the low quality of temporal satellite observations and the complexity of land surface properties. Moreover, the fusion of fine spatial resolution satellites (e.g., Landsat, Sentinel-2, and HLS) with high temporal resolution satellites (e.g., MODIS and VIIRS) has been proposed to improve both the spatial and temporal resolutions of the annual time series. For example, the fused Landsat-MODIS was produced to monitor crop progress and condition at field scales^38^ or the fused HLS-VIIRS time series was used to detect corn and soybean phenometrics over the US Corn Belt^39^. Although the temporal quality in the fused time series could be improved, the gaps still remain because persistent cloud cover or other factors mentioned above commonly exist in daily satellite observations (such as MODIS and VIIRS)^40,41^.

On the other hand, the PhenoCam network has been developed since 2008 that currently includes more than 700 sites to track the vegetation phenology across various ecosystems in North America and around the world using digital cameras (https://phenocam.nau.edu/)^42^. Unlike the optical satellite observations, the near-surface PhenoCam time series is comparatively unaffected by the cloud and atmospheric effects^2,43,44^. In addition, the network offers near-surface observations every 30 minutes from 4 am to 10 pm via the RGB (Red, Green, and Blue) imagery, which makes PhenoCam-derived time series almost continuous and gap-free. The RGB imagery provides a tool to calculate the Green Chromatic Coordinate (GCC), a proportional measure of the green band to the sum of all RGB channels, for observing vegetation dynamics in different ecosystems^45–47^. The PhenoCam-derived GCC time series typically presents a good correlation to time series of vegetation index from satellites^46,48,49^. Further, although the PhenoCam site is distributed locally, its imagery could capture diverse phenological events within a small area because the phenological variations exist in either heterogeneous or homogeneous vegetation types^50,51^. For instance, the phenological timing within one PhenoCam site could differ by approximately two weeks for greenup onset and almost a month for senescence onset^52^. As a result, the PhenoCam imagery in a single site is generally composed of many time series with diverse phenology developments. In other words, the PhenoCam imagery could potentially capture phenology development of most vegetation types in the corresponding region, which could be matched with the satellite-derived time series.

The evaluation of LSP detections is always challenging due to the lack of a high-quality reference dataset^53^. Generally, validation efforts have been conducted using three common approaches, including indirect comparison with model outputs or variables observed at the ground level^54,55^, specific-species observations at field plots and landscape scales^56–58^, and plant canopy observations from the PhenoCam network^23,45^. The previous validations have commonly shown a discrepancy of >10 days between field observations and LSP detections^53,59^. It is because the LSP measures the phenological timing of plant communities within a certain scale that could contain a large variation in the growing conditions and plant characteristics. Currently, PhenoCam is the best data source for validating LSP products; however, it is very hard to spatially match well with satellite pixels because of the differences in the viewing angle and canopy coverage^45,60–63^. Particularly, MODIS and VIIRS observations offer LSP detections at 500 m pixels, which could generate very large discrepancies with PhenoCam observations in highly heterogenous areas because of the spatial mismatch^53^. Alternatively, the finer spatial resolution LSP, such as detections from Landsat or HLS data, has been regularly used to validate coarser spatial resolution LSP (e.g., 500 m MODIS/VIIRS) to reduce the spatial mismatch. Still, the gaps in finer spatial resolution satellite time series are generally larger than those in the coarser satellite observations, which induce much larger uncertainties in LSP detections^10,52^. Thus, the comparison with finer resolution LSP (e.g., Landsat or HLS alone) could not be able to validate the coarse resolution LSP. Although the multi-source LSP (MSLSP) at 30 m derived from the HLS observations is able to provide phenological timing for North America, the noise and gaps in the HLS time series continue to be substantial challenges that negatively influence the realism of LSP detections and cause large uncertainties^10,52^.

Therefore, building on the proof-of-concept provided by Tran *et al*.^52^, we here presented a reference dataset of vegetation phenology development at 30 m pixels that was produced by bridging the temporal HLS observations with near-surface PhenoCam time series, which is HLS-PhenoCam LSP dataset (hereafter called HP-LSP). The HP-LSP dataset consists of 78 regions (each region covers 10 × 10 km^2^ with at least one PhenoCam site) across various plant functional types and climates in North America during 2019 and 2020, which leads to approximately 17 million samples with a spatial resolution of 30 m. The dataset includes two parts: (1) the 3-day synthetic gap-free HLS-PhenoCam EVI2 time series, and (2) spatially continuous and scalable phenometrics (greenup, maturity, senescence, and dormancy onsets) with up to three vegetation growing cycles. The HP-LSP dataset can be used to monitor the vegetation development in heterogeneous or homogenous areas, train models (e.g., machine learning models) for improving land use/land cover mapping, validate satellite-based phenological products (e.g., global MODIS and VIIRS LSP), develop phenological models, and analyze seasonality and climate changes on terrestrial ecosystems.

## Methods

### Study area

**Fig. 1:**
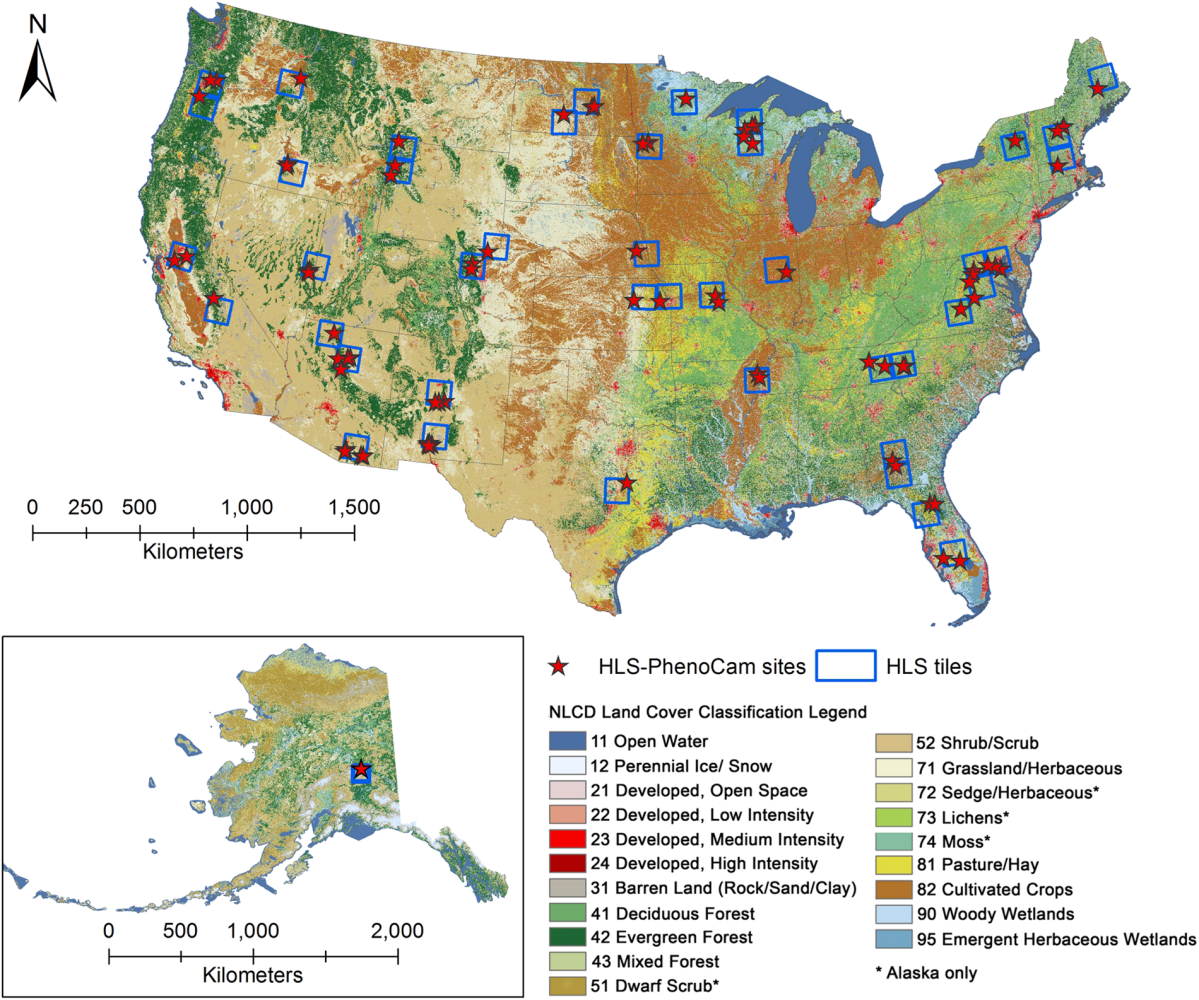
Geographical distribution of 78 regions included in the HLS-PhenoCam LSP (HP-LSP) dataset across various ecosystems in North America. Each region covers 10 × 10 km^2^ with at least one PhenoCam site. The background is the 30 m National Land Cover Database (NLCD) product displaying the land cover types (NLCD 2019 for CONUS and NLCD 2016 for Alaska).

We proposed 78 regions over various ecological systems, land cover types, and climate zones in North America to generate high-quality LSP for 2019 and 2020. The selection of regions was based on the sufficiency of Earth-observing satellite data and near-surface observations. Specifically, we selected all the HLS tiles with at least two standard PhenoCam sites that (1) are located within the HLS tile and an extension of the half tile size and (2) provide observations during 2018 and 2021. Then, we defined subsets (or regions) covering all PhenoCam sites inside each selected HLS tile, and the size of each region is 10 × 10 km^2^. As a result, 77 regions in the Contiguous US (CONUS) and 1 region in Alaska across 30 out of 50 US states were included in the high-quality HP-LSP dataset (Fig. 1 and Table S1).

### Datasets

This study mainly used satellite observations (HLS) and near-surface observations (PhenoCam). The HLS data are operationally produced from NASA by integrating OLI (Operational Land Imager) aboard the Landsat 8/9 satellites and the MSI (MultiSpectral Instrument) aboard the Sentinel-2A/2B satellites. The combination of these two measurements enables global observations of the Earth’s land surface at a moderate spatial resolution (30 m) and a high temporal resolution (every 2–3 days). The HLS provides a consistent surface reflectance seamed from both sensors (OLI and MSI) by applying a sequence of algorithms, including atmospheric correction, geometric resampling and common gridding, and nadir BRDF (bidirectional reflectance distribution function)-Adjusted Reflectance (NBAR) and spectral bandpass adjustment^64^. It is gridded into the UTM-based Military Grid Reference System (MGRS) with a tile coverage of 109.8 × 109.8 km^2^ with an overlap of 4,900 m on each side. The QA flags are also included in the HLS product indicating low-quality observations (snow/ice, cloud, cloud shadow, adjacent cloud, and cirrus clouds) and high-quality observations (others). As a result, 44 HLS tiles (Table S1 and Fig. 1) at version 1.4 between July 1, 2018 and July 1, 2021 were obtained for our study areas (https://hls.gsfc.nasa.gov/data/v1.4).

The near-surface observations were obtained from the PhenoCam network, which has been established since 2008 to monitor ecosystem dynamics using digital cameras mostly located in North America^45^. Deciduous forests, grasslands, and evergreen forests are the three most common vegetation types captured by PhenoCam cameras, while other vegetation types (e.g., agriculture, shrubs, tundra, and wetland) are less represented. Over 60 million RGB pictures publicly available in the archive provide a rich data resource for evaluating LSP products^16,20,51^, developing phenology models^42,65^, understanding the biophysical mechanism of vegetation^66^, and assessing the impacts of the climate change on terrestrial ecosystems^43^. This study extracted all JPEGs in the archive of 146 PhenoCam sites (Table S1) from the PhenoCam network images between July 1, 2018 and July 1, 2021 from 9 am to 5 pm (https://phenocam.nau.edu/).

### Generation of the 30 m HP-LSP dataset

The fusion of temporal HLS observations with PhenoCam time series for improving the accuracy of LSP detections has been demonstrated in detail in the previous study^52^; thus, it was briefly described as the following.

### Calculation of vegetation index time series for HLS and PhenoCam data

The HLS EVI2 (two-band enhanced vegetation index) was selected for detecting LSP across 78 10 × 10 km^2^ regions in this study. It is because (1) EVI2 is less sensitive to the background reflectance and saturation in densely vegetated areas^67^, which makes it better in detecting phenological timing compared to the *in situ* PhenoCam observations^53,60^ and (2) the EVI2 time series has been widely used to generate operational LSP products from continental to global scales^9,10,68–70^. Specifically, the QA flags in the HLS product were first used to select only high-quality observations but remove low-quality observations. The 3-day composite of HLS EVI2 time series for a pixel was then generated by selecting or averaging high-quality observations every three days if more than one observation was available. The 3-day HLS EVI2 value was assigned as a fill value (or a gap) if (1) no any high-quality observations exist within the 3-day window, (2) it is greater than 90% of co-located 3-day NDVI (normalized difference vegetation index) value or greater than 110% of any 3-day EVI2 values in the preceding and succeeding one-month period (indication of abiotic noise), or (3) co-located 3-day NDVI is less than 3-day NDWI (normalized difference water index) (indication of residual contamination from cloud, snow, or land surface moisture).

The PhenoCam GCC (green chromatic coordinate) time series was fully extracted using a newly developed framework instead of using the limited GCC time series available in each site from the PhenoCam dataset version v2.0^52^. This framework divides PhenoCam imagery into 10 × 10 grids of equal size in each single PhenoCam site. The resultant grids could reflect considerably different phenological behaviors for either homogeneous or heterogeneous vegetation types captured by the PhenoCam camera in a small area. It is because the temporal patterns of vegetation growth could vary largely within a very small vegetation canopy area with the differences in the plant characteristic and microclimate^38,50,70,71^. Particularly, GCC was calculated for each individual grid on the half-hourly PhenoCam images, which was then aggregated to a 3-day GCC composite by selecting the 90th percentile value. The composite ensures a gap-free and high-quality GCC time series because it can remove high-frequency noise and minimize the negative impacts of power outages and unfavorable weather conditions (i.e., snow, rain, and fog)^72^. The 3-day GCC composite also makes it temporally consistent with the 3-day HLS EVI2 time series. As a result, for every 10 × 10 km^2^ region selected inside a HLS tile, a diverse collection of grid-based PhenoCam GCC time series was established from all PhenoCam sites located within one and a half HLS tile size. If several 10 × 10 km^2^ regions are defined within a HLS tile, they use a mutual collection of PhenoCam GCC time series for that HLS tile.

### Fusion of HLS and PhenoCam vegetation index time series

The synthetic gap-free HLS-PhenoCam time series was generated by fusing the HLS time series with the PhenoCam time series based on the following facts. (1) The 3-day HLS EVI2 time series always contains a large number of fill values (gaps), which significantly affects the accuracy of phenological detections^10,16^. (2) The HLS EVI2 value in a pixel is the linear contributions from green vegetation, colored vegetation, and exposed surface background (mainly bare soil and rock)^73–75^, in which a similar proportional composition could be found from a PhenoCam grid in the surrounding area. (3) The HLS EVI2 time series is highly temporal correlated with the corresponding PhenoCam GCC time series in the same area^48,49^ and their phenological transition dates are closely comparable^51,53,59,76^. (4) The HLS EVI2 time series in a pixel is likely to match well with a grid-based GCC temporal shape in a local area where several PhenoCam sites are available, although their growing seasons and greenness magnitude could be inconsistent. (5) A grid-based PhenoCam GCC time series could be geometrically and temporally scalable to the equivalent EVI2 time series in a HLS pixel if their fractions of surface components are similar despite the fact that their spatial coverages and viewing geometry are different.

Practically, the HLS EVI2 time series for a given pixel was compared with each grid-based GCC time series from the PhenoCam GCC collection using the spatiotemporal shape-matching model (SSMM) described in the Eqs. 1, 2^76^. The geometric mean functional regression (GMFR) was integrated into the SSMM to calculate mean squared deviation (MSD) and correlation coefficient (R) between raw HLS and predicted HLS values from Eq. 1. Ultimately, the PhenoCam GCC time series with the smallest MSD and highest R was selected to fuse with the HLS EVI2 time series. If the HLS EVI2 time series and the best comparable GCC time series are poorly correlated (R ≤ 0.6 and p > 0.02), the fusion was not performed.

1 $$\overline{HLS}\left(t\right)=a\times PhenoCam\left(T\right)+b$$

2 $$T=\lambda \times \left(t+\beta \right)$$

where *t* and *T* are the time in the number of days; $\overline{HLS}\left(t\right)$ and *PhenoCam* (*T*) are predicted HLS EVI2 values at the time *t* and PhenoCam GCC values at the time *T*, respectively; *a, b, λ*, and *β* are four scaling factors. In which, 0.9 ≤ *λ* ≤ 1.1 with a 0.05 increment reflects the ratio of growing season length between HLS and PhenoCam. −30 ≤ *β* ≤ 30 days with a 3-day increment indicates the seasonal shift between HLS and PhenoCam phenology^16^. *a* and *b* are the slope and intercept in the linear function in the Eq. 1.

Once the most comparable GCC time series was selected from the collection of PhenoCam GCC time series, its optimal scaling factors *a, b, λ*, and *β* were recalled to predict the EVI2 values for all the gaps in the given HLS EVI2 time series using Eqs. 1, 2. Using this approach, a synthetic gap-free HLS-PhenoCam EVI2 time series was generated at 30 m spatial resolution.

### Detection of phenometrics from the synthetic gap-free HLS-PhenoCam EVI2 time series

The Hybrid Piecewise Logistic Model (HPLM) based Land Surface Phenology Detection (LSPD) algorithm has been successfully employed to produce NASA operational MODIS and VIIRS LSP products^15,68^ and described in detail in previous studies^4,25^. Thus, it was chosen to detect phenological dates from the synthetic HLS-PhenoCam time series with five primary steps. First, the background EVI2 value was calculated by averaging EVI2 values that are smaller than the 10th percentile of the sorted HLS-PhenoCam time series. Second, the HLS-PhenoCam time series was smoothed to further reduce potential noise and irregular variations by using Savitzky-Golay filter and moving average and median methods. Third, the smoothed HLS-PhenoCam time series was divided into greenup and senescence phases by identifying the slope changes using a five 3-day window. Fourth, HPLM was applied to reconstruct the greenup and senescence trajectories of vegetation growth cycle^25^:

3 $$EVI2\left(t\right)=\left\{\begin{array}{cc}\frac{{c}_{1}}{1+{e}^{{a}_{1}+{b}_{1}\times t}}+EVI{2}_{b} & Favorable\;growth\;condtion\;\left(a\right)\\ \frac{{c}_{2}+d\times t}{1+{e}^{{a}_{2}+{b}_{2}\times t}}+EVI{2}_{b} & Vegetation\;stress\;condtion\;\left(b\right)\end{array}\right.$$

where *t* is time in the day of year (DOY), *a* is related to the vegetation growth period, *b* is associated with the rate of plant leaf development, *c* is the amplitude of EVI2 variation, *d* is a vegetation stress factor^32,77^, and *EVI2*_*b*_ is the background (dormant season) value.

Finally, four key phenological transition dates or phenometrics (greenup, maturity, senescence, and dormancy onsets) were detected by calculating the local extremes of curvature change rate on the HPLM reconstructed EVI2 time series.

## Data Records

**Fig. 2:**
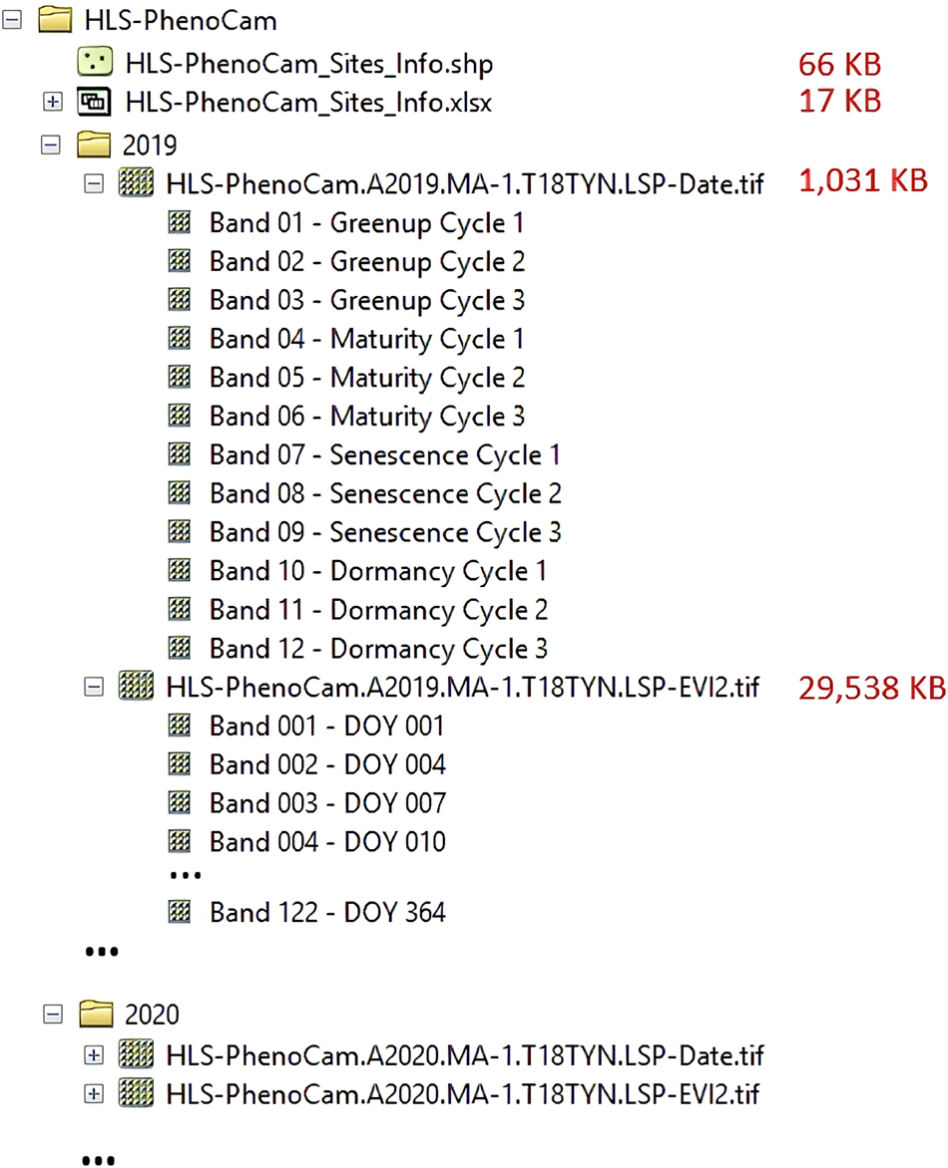
The structure of the HLS-PhenoCam LSP (HP-LSP) dataset stored in the archive. Viewing via ArcMap Catalog.

The 30 m HP-LSP dataset^78^ is permanently and publicly available through the NASA’s Oak Ridge National Laboratory Distributed Active Archive Center (ORNL DAAC) (10.3334/ORNLDAAC/2248), which consists of two main folders corresponding to the years 2019 and 2020, respectively. In addition, an Excel file and a shapefile containing the information of the HP-LSP dataset (such as Site ID, centered geographical location, primary vegetation, HLS tile coverage, and PhenoCam sites selected for fusion) were also included in the dataset. Particularly, for each yearly HLS-PhenoCam region, two Geotiff files consume 30 MB of storage, including the LSP (1 MB) and synthetic EVI2 time series (29 MB) (Fig. 2). The filenames are structured as follows:

*HLS-PhenoCam.AYYYY.SiteID.HLStile.LSPdata.tif*

Here, *HLS-PhenoCam* is the short name of the product (HLS-PhenoCam land surface phenology); *AYYYY* (e.g., A2019) is the year of LSP acquisition; *SiteID* is combined by short state name and ID within that state (e.g., MA-1, please check Table S1); *HLStile* (e.g., T19TEL) is the HLS tile covers the HLS-PhenoCam region; *LSPdata* could be the *LSP-Date* (four key phenological transition dates) and *LSP-EVI2* (3-day synthetic gap-free HLS-PhenoCam EVI2 time series); and*.tif* is Geotiff format.

Currently, the *LSP-Date* file stored 12 bands corresponding to four key onsets (greenup, maturity, senescence, and dormancy) with up to three cycles. The *LSP-EVI2* file stored 122 bands corresponding to 122 3-day synthetic gap-free HLS-PhenoCam EVI2 that starts from January 1 to December 31 of the year (Fig. 2). The unit of *LSP-Date* is the day of year (DOY) and the *LSP-EVI2* values range from −10,000 to 10,000. The data type is 16-bit signed integer with the *nodata* value set as 32767 for both *LSP-Date* and *LSP-EVI2*.

**Fig. 3:**
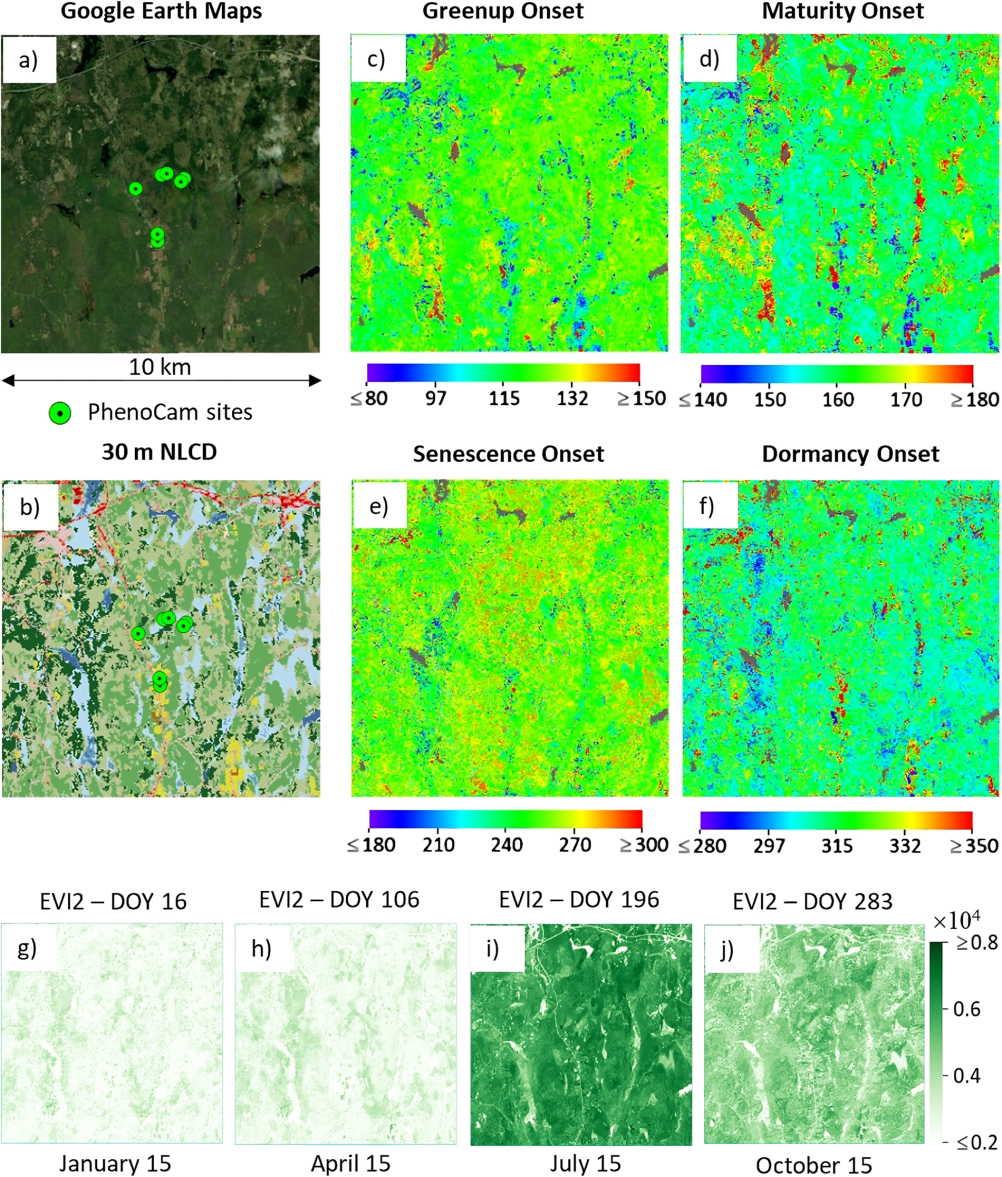
An example of spatial patterns of HP-LSP dataset at the MA-1.T18TYN (centered location at 42.53°N and 72.18°W). (**a**) is the Google Earth Map, (**b**) is the 30 m NLCD with a similar legend in Fig. 1, (**c**–**f**) are four key phenometrics detected from the synthetic HLS-PhenoCam EVI2 time series (greenup, maturity, senescence, and dormancy onset, respectively), and (**g**–**j**) are HLS-PhenoCam EVI2 on DOY 16 (January 15), DOY 106 (April 15), DOY 196 (July 15), and DOY 283 (October 15), respectively. The gray color in phenometrics is water.

Figure 3 shows an example of the HP-LSP dataset stored in the archive for site MA-1.T18TYN (centered location at 42.53°N and 72.18°W). We displayed the spatial patterns of four key phenometrics (greenup, maturity, senescence, and dormancy onsets) in the cycle 2 (c-e, respectively) and four synthetic EVI2 bands on DOYs 16, 106, 196, and 283 (middle of January, April, July, and October, respectively).

## Technical Validation

We here conducted three primary assessments to validate the HP-LSP dataset. First, the generation of the synthetic gap-free HLS-PhenoCam EVI2 time series was evaluated for five typical plant functional types in North America. We randomly selected five pixels nearby five PhenoCam sites, which represent deciduous forest, evergreen forest, agriculture, grass, and shrub, to demonstrate the superiority of the dataset produced by fusing the HLS time series with the temporal shape of near-surface PhenoCam observations. Second, the HLS-PhenoCam phenometrics detected from the synthetic gap-free HLS-PhenoCam time series were validated using the phenological timing obtained from the near-surface observations after performing the spatial match. In this process, we visually matched PhenoCam imagery, Google Earth map, and HLS pixels and then defined the independent PhenoCam ROIs (region of interest) across 78 HLS-PhenoCam regions that were clearly distinguishable from the corresponding HLS pixels. The phenometrics in the spatially matched ROIs were extracted from PhenoCam imagery for validating the overall quality of HLS-PhenoCam phenology. Lastly, the HLS-PhenoCam phenometrics were closely examined for five plant functional types to assess the accuracy of phenological detections on each ecosystem. The details of these three assessments were presented as followings.

### Assessment of generating synthetic gap-free HLS-PhenoCam time series by fusing HLS EVI2 time series with PhenoCam GCC time series

**Fig. 4:**
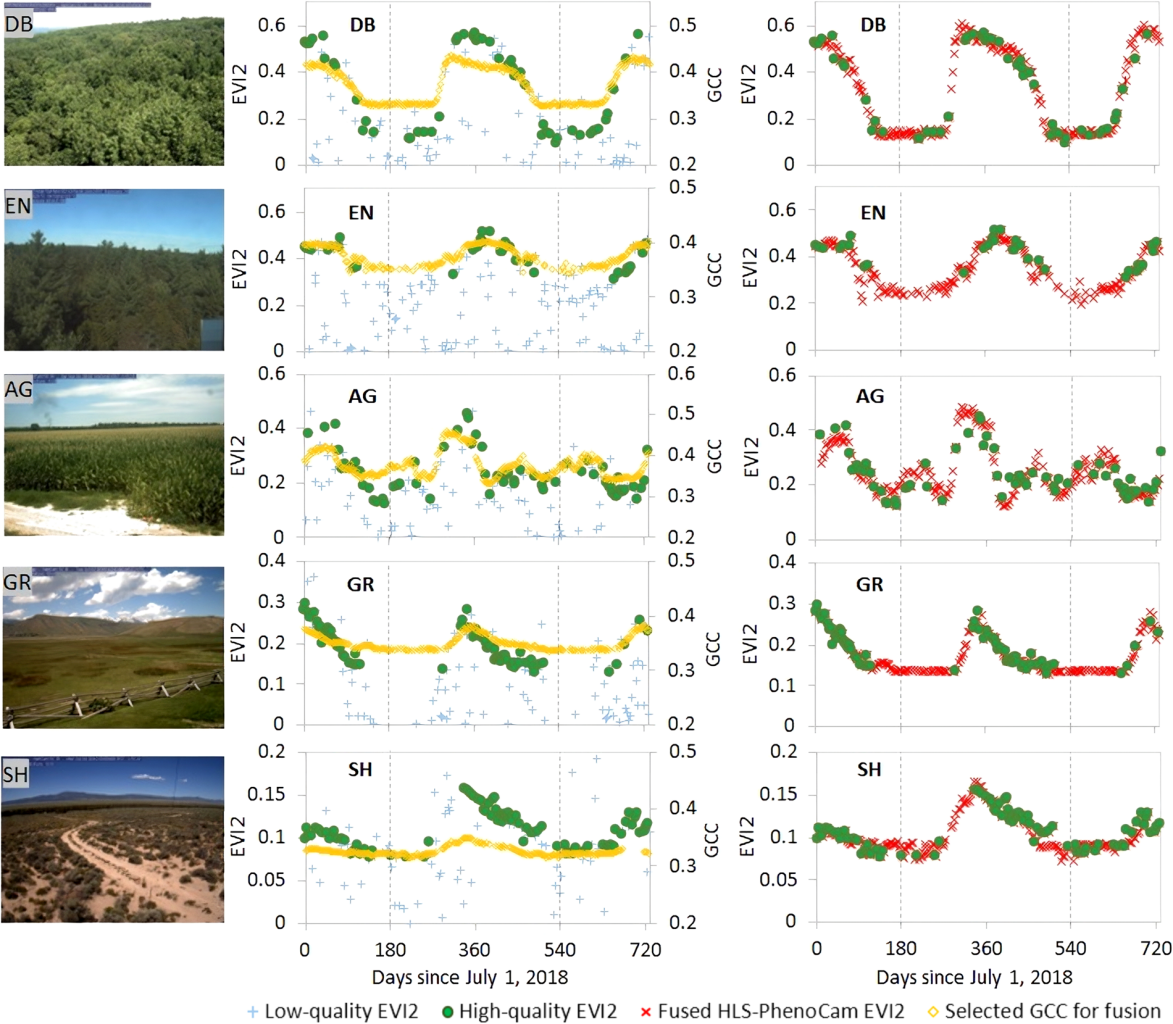
Illustration of fusing HLS EVI2 time series with PhenoCam GCC time series to generate 30 m synthetic gap-free HLS-PhenoCam time series for five pixels of deciduous forest (DB), evergreen forest (EN), agriculture (AG), grass (GR), and shrub (SH) nearby PhenoCam sites NEON.D02.SCBI.DP1.00033, howland1, arsgacp1, nationalelkrefuge, nevcanspg1a, respectively. The dates start from July 1, 2018 to July 1, 2020, with the main year 2019 separated by two vertical dash lines.

Figure 4 shows the generation of the 30 m synthetic gap-free HLS-PhenoCam EVI2 time series for five typical plant functional types across various climate regions in North America, including deciduous forest, evergreen forest, agriculture, grass, and shrub. The HLS pixels for deciduous forests and evergreen forests were selected in the temperate regions of the Northeastern US and nearby the PhenoCam sites *NEON.D02.SCBI.DP1.00033* (camera pole located at 38.893°N and 78.139°W) and *howland1* (camera pole located at 45.204°N and 68.740°W), respectively; the HLS pixel for agriculture or croplands was selected in the humid area of the Southeastern US and nearby PhenoCam site *arsgacp1* (camera pole located at 31.511°N and 83.618°W); and the HLS pixels for grasses and shrubs were selected in the semiarid areas of the Western US and nearby the *nationalelkrefuge* (camera pole located at 43.489°N and 110.738°W) and *nevcanspg1a* (camera pole located at 38.925°N and 114.408°W), respectively. Generally, the raw HLS EVI2 time series were very noisy and most of the observations were of low quality, while the high-quality observations were sparsely distributed along the trajectory of vegetation growing cycles. In all five samples, the high-quality observations in 2019 were only 23%, 11%, 22%, 19%, and 28%, respectively. Thus, the phenometrics derived from the HLS data alone could be highly impacted due to the limited number of high-quality observations across all ecological regions. In contrast, the selected GCC time series were gap-free and their temporal shapes were highly correlated with the high-quality HLS EVI2 time series. In particular, the HLS EVI2 time series for the deciduous forest, evergreen forest, grass, and shrub pixels were able to separately match with their optimal GCC time series with a very strong correlation (R > 0.9 and p < 0.01), which was similar for the agriculture sample (R = 0.88 and p < 0.01). Further, the synthetic HLS-PhenoCam EVI2 time series were generated by replacing low-quality EVI2 values in the raw HLS time series with the fused HLS-PhenoCam EVI2 values using the SSMM (Eqs. 1, 2). Although HLS EVI2 time series is not always able to present distinctive seasonality because of various impacts, such as in some evergreen forest and agriculture pixels, the synthetic gap-free HLS-PhenoCam EVI2 time series effectively imitated seasonal dynamics of vegetation growths and multiple growing cycles.

### Evaluation of HLS-PhenoCam phenometrics with the near-surface PhenoCam observations

The HLS-PhenoCam phenometrics were evaluated using the independently-generated near-surface PhenoCam observations. A set of HLS pixels from 78 HLS-PhenoCam regions were spatially matched with the PhenoCam ROIs by visually matching with the PhenoCam imagery, Google Earth map, and HLS pixel grids. Accordingly, we selected 125 HLS pixels that were linked with 116 out of 146 PhenoCam sites, where each PhenoCam site matched up with one or two HLS pixels. On the other hand, 30 out of 146 PhenoCam sites were hard to match with HLS pixels or outside the 10 × 10 km^2^ HLS-PhenoCam regions (only used for the fusion process). To be specific, the independently appropriate PhenoCam ROIs selected for the validation were manually defined and not a circulation of repeatedly using the PhenoCam grid in the fusion process^52^. The validation reliability is verified based on the following facts. (1) One 30 m HLS pixel usually covers a large proportion of PhenoCam imagery and could include several PhenoCam grids with different phenological transition dates, while the optimal GCC time series is only selected from one grid for fusing with HLS EVI2 time series. (2) The collection of PhenoCam GCC time series is created from all PhenoCam sites in the areas regardless of geolocation. The SSMM iteratively goes through the PhenoCam GCC collection and very likely selects an optimal grid-based PhenoCam GCC time series from a PhenoCam site at a different location. (3) The SSMM only focuses on the temporal shape of two time-series inputs. Each grid-based PhenoCam GCC time series in the collection was scaled up to match and fuse with the HLS EVI2 time series, while ignoring the differences in seasonal shift, magnitude of greenness, and vegetation growth duration.

The near-surface phenological transition dates were obtained from the corresponding ROI-based PhenoCam GCC time series using the HPLM-LSPD algorithm. The derived PhenoCam phenometrics were then used to evaluate the HLS-PhenoCam phenometrics in 2019 and 2020 by calculating their correlation coefficient (R^2^), root mean squared error (RMSE), mean absolute difference (MAD), and mean systematic bias (MSB).

**Fig. 5:**
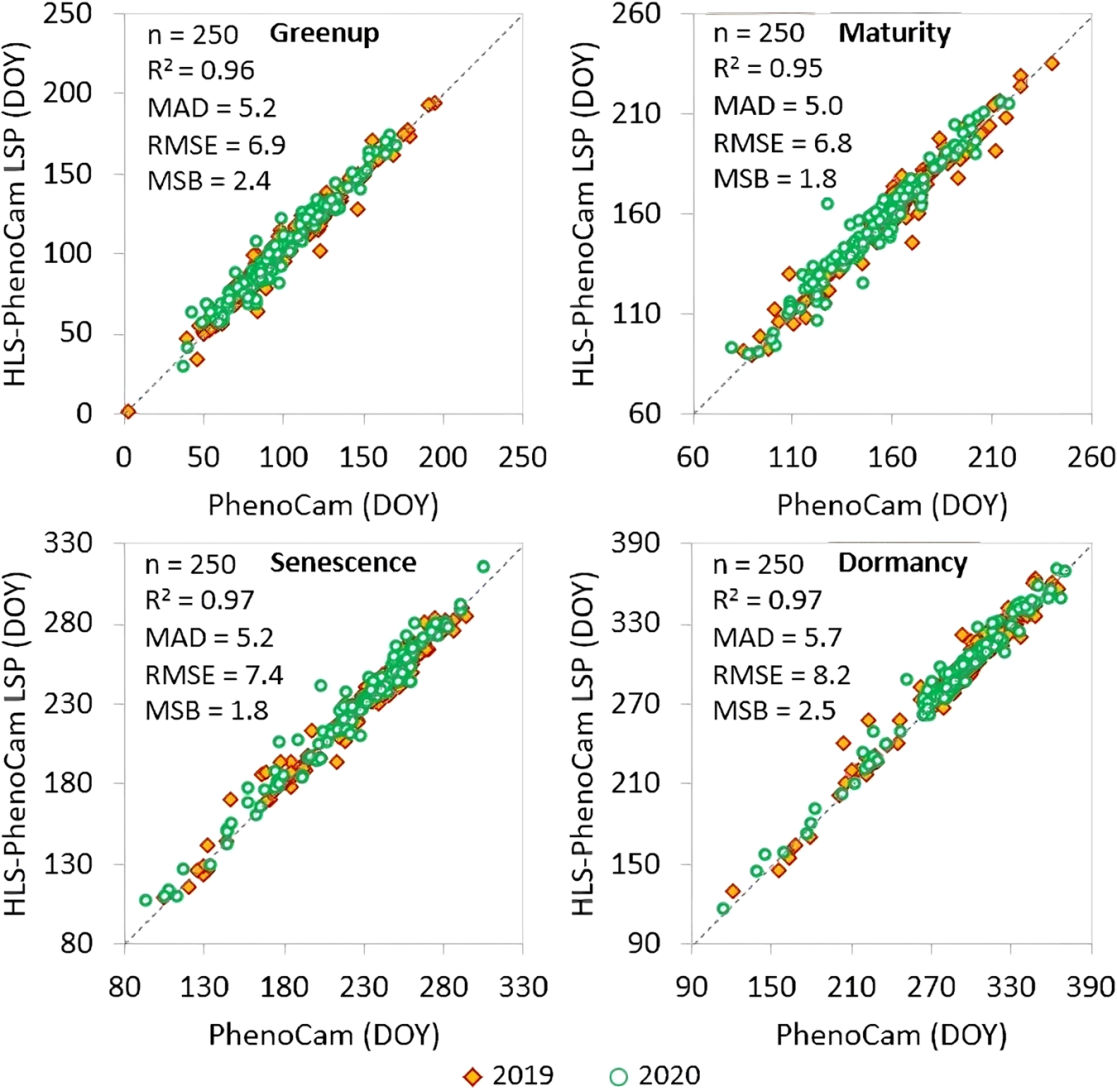
Overall comparison of HLS-PhenoCam phenometrics with near-surface Phenocam observations for four key phenological transition dates (greenup, maturity, senescence, and dormancy onsets) in 2019 and 2020. The dashed line indicates the 1:1 agreement.

Figure 5 displays the scatter plots of phenometrics derived from the synthetic gap-free HLS-PhenoCam EVI2 time series and the near-surface PhenoCam observations based on 125 HLS pixel samples and 116 PhenoCam sites that were spatially linked to each other. The HLS-PhenoCam phenometrics were very close to the near-surface phenology for the years 2019 and 2020 in all four onsets (greenup, maturity, senescence, and dormancy). The statistical analyses showed a strong correlation between HLS-PhenoCam phenometrics and near-surface PhenoCam observations: the R^2^ ≥ 0.95, MAD ≤ 5 days, RMSE ≤ 8 days, and MSB ≤ 2 days. It should be noted that their differences are much smaller than previous studies^10,20,56,79^.

**Fig. 6:**
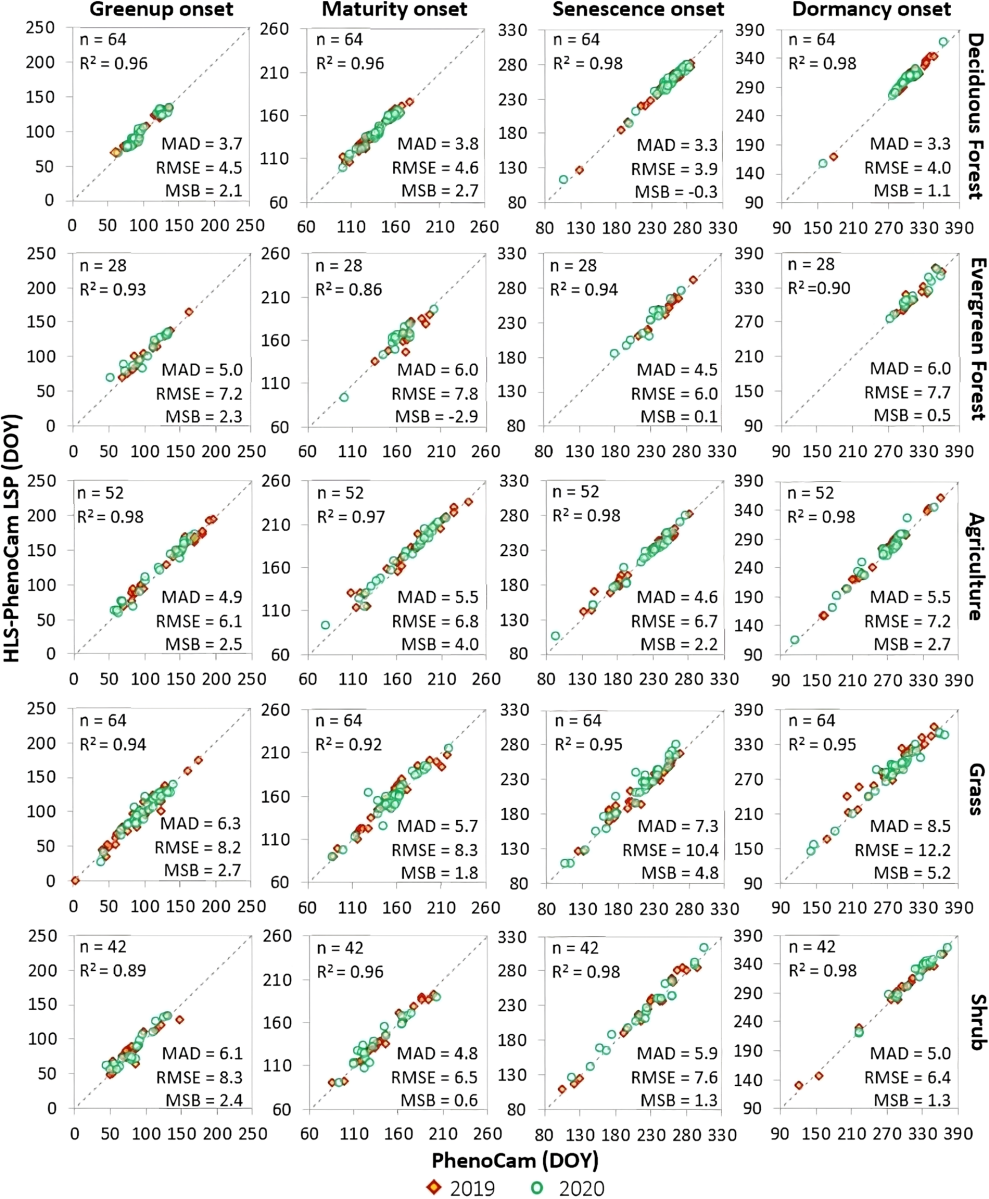
Comparison of HLS-PhenoCam phenometrics with near-surface Phenocam observations for four key phenological transition dates for five typical plant functional types in North America in 2019 and 2020. This figure is arranged by rows and columns. The rows represent five typical plant functional types, including deciduous forest, evergreen forest, agriculture, grassland, and shrub, respectively. The columns represent four key phenological transition dates, including greenup onset, maturity onset, senescence onset, and dormancy onset, respectively. The vertical axis is the HLS-PhenoCam phenometrics and the horizontal axis is PhenoCam phenometrics for all panels. The dashed line indicates the 1:1 agreement.

Figure 6 displays the relationship between the HP-LSP and PhenoCam observations based on five typical plant functional types in North America, including the deciduous forest, evergreen forest, agriculture, grass, and shrub, respectively. In general, the HLS-PhenoCam phenological detections had a good agreement for all five plant functional types compared to those derived from the near-surface PhenoCam observations. The deciduous forest (26% of total samples) obtained the strongest correlation and very close to the PhenoCam observations with R^2^ ≥ 0.96, MAD ≤ 3 days, RMSE ≤ 4 days, and MSB ≤ ±2 days. The evergreen forest (11% of total samples), agriculture (21% of total samples), and shrub (17% of total samples) were at the same level and strongly correlated with PhenoCam observations: the R^2^ ≥ 0.86, MAD ≤ 6 days, RMSE ≤ 8 days, and MSB ≤ ±4 days. Besides, the statistical relationship for grass pixels (25% of total samples) was highly associated with PhenoCam in the greenup phase (R^2^ ≥ 0.92, MAD ≤ 6 days, RMSE ≤ 8 days, and MSB ≤ 2 days), while a slightly weaker relationship was noticed in the senescence phase (R^2^ ≥ 0.95, MAD ≤ 8 days, RMSE ≤ 12 days, and MSB ≤ 5 days).

## Usage Notes

The authors would like to have two notes for users. First, although we store phenometrics (*LSP-Date*) with three cycles, the main growing cycle is usually stored in cycle 2 and it could be stored in cycle 3 if there is a smaller cycle occurs prior to the main cycle (e.g., cover crops). Thus, the users should consider which phenometric band (or cycle) is used for their analyses. Second, the 3-day synthetic HLS-PhenoCam EVI2 time series (*LSP-EVI2*) could contain very short gaps in the main year period if there is a gap in the PhenoCam GCC time series selected for fusion.

Further, we currently only generated the reference dataset of land surface phenology over the USA because of the availability of PhenoCam Network. The PhenoCam sites have rapidly increased across the globe during recent years. Although many sites are owned by private units, such as in China and Australia, some publicly accessible digital cameras are also available in Europe and Japan. On the other hand, HLS data are operationally produced in NASA across the globe. Therefore, users can extend the reference dataset to other regions beyond the USA using the approach provided in the study once they are able to obtain Phenocam observations in a region of interest.

### Supplementary information

HP-LSP dataset info

## Data Availability

The computer code and instructions be obtained through a public repository at https://github.com/khuonghtran/SSMM. Examples of input data for the code can be obtained from https://openprairie.sdstate.edu/global_land_surface_season_data/5/.
