# Supplementary material for: HP-LSP: A reference of land surface phenology from fused Harmonized Landsat and Sentinel-2 with PhenoCam data
Source: Sci Data. 2023 Oct 11;10:691. doi: 10.1038/s41597-023-02605-1 (PMC10567776; doi:10.1038/s41597-023-02605-1)
Supplement: Supplementary file 1 — HP-LSP dataset info [file 41597_2023_2605_MOESM1_ESM.docx]

**HP-LSP: A reference of land surface phenology from fused Harmonized Landsat and Sentinel-2 with PhenoCam data**

Khuong H. Tran^1^, Xiaoyang Zhang^1⁎^, Yongchang Ye^1^, Yu Shen^1^, Shuai Gao^1^, Yuxia Liu^1^ & Andrew Richardson^2,3^

1. Geospatial Sciences Center of Excellence, Department of Geography & Geospatial Sciences, South Dakota State University, Brookings, SD 57007, USA

2. School of Informatics, Computing, and Cyber Security, Northern Arizona University, Flagstaff, AZ 86011, USA

3. Center for Ecosystem Science and Society, Northern Arizona University, Flagstaff, AZ 86011, USA

*Corresponding author(s): Xiaoyang Zhang ([xiaoyang.zhang@sdstate.edu](mailto:xiaoyang.zhang@sdstate.edu))

Table of contents

1. Supplementary Table S1

# Supplementary Information

Table S1. All 10 × 10 km^2^ regions are included in the high-quality HLS-PhenoCam LSP dataset. The first two letters of Site ID indicate the US state and territory abbreviations. The primary vegetation types include DB – Deciduous forests, EN – Evergreen forests, GR – Grass, AG – Agriculture, and SH – Shrub.

| **No.** | **Site ID** | **Centered location** | | **Primary vegetation** | **HLS tile** | **PhenoCam sites selected for fusion with HLS data** | |
| --- | --- | --- | --- | --- | --- | --- | --- |
|  |  | **Lat** | **Lon** |  |  | **Inside HLS tile and Covered by HLS-PhenoCam region** | **Inside extension of HLS tile and only used for fusion** |
| 1 | ME-1 | 45.20 | -68.74 | EN, DB | T19TEL | howland1 howland2 |  |
| 2 | NH-1 | 44.06 | -71.29 | DB, EN | T18TYP | bartlettir |  |
| 3 | NH-2 | 43.94 | -71.70 | DB, EN |  | hubbardbrook |  |
| 4 | NY-1 | 43.98 | -74.23 | EN, DB | T18TWP | arbutuslakeinlet goodnow |  |
| 5 | MA-1 | 42.53 | -72.18 | DB, EN & GR | T18TYN | harvardfarmnorth harvardfarmsouth harvardbarn harvardhemlock harvardhemlock2 harvardhemlock2 NEON.D01.HARV.DP1.00033 | macleish worcester |
| 6 | MD-1 | 39.14 | -77.21 | GR, DB | T18SUJ | nist |  |
| 7 | MD-2 | 39.03 | -76.84 | AG |  | arsope3ltar |  |
| 8 | MD-3 | 38.89 | -76.56 | DB |  | NEON.D02.SERC.DP1.00033 |  |
| 9 | VA-1 | 39.03 | -78.04 | DB, EN | T18STJ | NEON.D02.BLAN.DP1.00033 |  |
| 10 | VA-2 | 38.89 | -78.14 | DB |  | NEON.D02.SCBI.DP1.00033 |  |
| 11 | VA-3 | 38.62 | -78.35 | DB, EN | T18STH | shenandoah |  |
| 12 | VA-4 | 37.92 | -78.27 | DB, EN |  | pace |  |
| 13 | VA-5 | 37.57 | -79.09 | EN, DB & GR | T17SPB | sweetbriar sweetbriargrass sweetbriarclearcut |  |
| 14 | NC-1 | 35.61 | -82.44 | DB, EN | T17SLV | warrenwilson | pace NEON.D07.GRSM.DP1.00033 |
| 15 | NC-2 | 35.60 | -82.55 | DB, EN |  | unca |  |
| 16 | TN-1 | 35.69 | -83.50 | DB, EN | T17SKV | NEON.D07.GRSM.DP1.00033 |  |
| 17 | TN-2 | 35.96 | -84.28 | DB, EN |  | NEON.D07.ORNL.DP1.00033 |  |
| 18 | GA-1 | 31.73 | -83.74 | AG | T17SKR | arsgacp3 arsgacp4 | arsgacp1 NEON.D03.JERC.DP1.00033 |
| 19 | GA-2 | 31.51 | -83.62 | AG | T17RKQ | arsgacp1 | arsgacp3 arsgacp4 NEON.D03.JERC.DP1.00033 |
| 20 | FL-1 | 29.74 | -82.22 | EN | T17RLN | austincary |  |
| 21 | FL-2 | 29.69 | -81.99 | EN |  | NEON.D03.OSBS.DP1.00033 |  |
| 22 | FL-3 | 27.18 | -81.21 | AG & GR | T17RML | archboldbahia archboldavirx archboldavir archboldpnotx archboldpnot | NEON.D03.DSNY.DP1.00033 |
| 23 | FL-4 | 27.38 | -81.95 | SH |  | ufona |  |
| 24 | WI-1 | 45.49 | -89.59 | DB, EN | T15TYL | NEON.D05.STEI.DP1.00033 NEON.D05.TREE.DP1.00033 | lostcreek |
| 25 | WI-2 | 45.81 | -90.08 | DB |  | willowcreek |  |
| 26 | WI-3 | 46.08 | -89.98 | GR | T15TYM | lostcreek | willowcreek |
| 27 | MI-1 | 46.24 | -89.35 | EN, DB |  | sylvania |  |
| 28 | MI-2 | 46.23 | -89.54 | DB, EN |  | NEON.D05.UNDE.DP1.00033 |  |
| 29 | IL-1 | 40.04 | -88.20 | AG | T16TCK | uiefmaize2 uiefsorghum |  |
| 30 | MN-1 | 47.50 | -93.45 | EN | T15TVN | spruceA0EMI spruceA0P07 spruceA0P21 |  |
| 31 | MN-2 | 45.68 | -95.80 | AG | T14TQR | arsmnswanlake1 |  |
| 32 | MN-3 | 45.63 | -96.13 | AG |  | arsmorris1 arsmorris2 |  |
| 33 | MO-1 | 39.23 | -92.15 | AG | T15SWD | goodwaterbau goodwater | missouriozarks |
| 34 | MO-2 | 38.95 | -92.00 | GR |  | tuckerprairie |  |
| 35 | AR-1 | 35.73 | -90.04 | AG | T15SYV | usof5 usof6 |  |
| 36 | AR-2 | 35.89 | -90.14 | AG |  | manilacotton |  |
| 37 | ND-1 | 46.78 | -100.95 | AG & GR | T14TLS | mandanh5 NEON.D09.NOGP.DP1.00033 mandani2 |  |
| 38 | ND-2 | 47.16 | -99.11 | GR | T14TMT | NEON.D09.DCFS.DP1.00033 |  |
| 39 | ND-3 | 47.13 | -99.24 | GR, DB & EN |  | NEON.D09.WOOD.DP1.00033 NEON.D09.PRPO.DP1.20002 |  |
| 40 | NE-1 | 41.14 | -96.46 | AG | T15TTF | mead1 mead2 mead3 meadpasture | ninemileprairie |
| 41 | KS-1 | 39.06 | -95.19 | GR, DB | T15SUD | kansas NEON.D06.UKFS.DP1.00033 |  |
| 42 | KS-2 | 39.11 | -96.59 | GR, DB | T14SQJ | NEON.D06.KONZ.DP1.00033 NEON.D06.KING.DP1.20002 NEON.D06.KONA.DP1.00033 | kansas NEON.D06.UKFS.DP1.00033 |
| 43 | TX-1 | 31.48 | -96.89 | GR, AG | T14RPV | tworfaa tworfpr tworfta | twosfpr |
| 44 | WY-1 | 44.95 | -110.59 | EN, GR | T12TWQ | NEON.D12.YELL.DP1.00033 NEON.D12.BLDE.DP1.20002 |  |
| 45 | WY-2 | 43.92 | -110.58 | GR | T12TWP | grandteton |  |
| 46 | WY-3 | 43.49 | -110.74 | GR |  | nationalelkrefuge |  |
| 47 | CO-1 | 40.82 | -104.75 | AG, GR | T13TEF | cperagm cpertgm cperheavy cperuvb NEON.D10.CPER.DP1.00033 |  |
| 48 | CO-2 | 40.28 | -105.55 | EN | T13TDE | NEON.D10.RMNP.DP1.00033 | cperagm cpertgm cperheavy cperuvb NEON.D10.CPER.DP1.00033 |
| 49 | CO-3 | 40.05 | -105.57 | GR & SH |  | niwot3 NEON.D13.NIWO.DP1.00033 |  |
| 50 | NM-1 | 34.44 | -106.25 | SH | T13SCU | usmpj |  |
| 51 | NM-2 | 34.39 | -106.53 | GR |  | sevpjrm13 |  |
| 52 | NM-3 | 34.35 | -106.71 | GR & SH |  | sevilletanewgrass sevilletagrass sevilletashrub sevMRME1S sevMRME10L |  |
| 53 | NM-4 | 32.58 | -106.63 | SH | T13SCS | jerbajada |  |
| 54 | NM-5 | 32.63 | -106.77 | GR & SH |  | jernwern jernort |  |
| 55 | NM-6 | 32.59 | -106.85 | GR & SH |  | ibp NEON.D14.JORN.DP1.00033 |  |
| 56 | NM-7 | 32.52 | -106.80 | SH |  | jersand |  |
| 57 | WA-1 | 46.78 | -117.08 | AG | T11TMM | cafcookeastltar01 cafcookwestltar01 cafboydnorthltar01 cafboydsouthltar01 |  |
| 58 | WA-2 | 45.82 | -122.33 | EN | T10TER | NEON.D16.WREF.DP1.00033 | silverton |
| 59 | WA-3 | 45.76 | -121.95 | EN |  | NEON.D16.ABBY.DP1.00033 |  |
| 60 | OR-1 | 45.00 | -122.69 | AG | T10TEQ | silverton | oregonYP |
| 61 | ID-1 | 43.06 | -116.75 | SH | T11TNH | arsgreatbasinltar177 |  |
| 62 | ID-2 | 43.17 | -116.71 | SH |  | arsgreatbasinltar098 |  |
| 63 | NV-1 | 39.01 | -114.31 | EN | T11SQD | nevcansnk3a |  |
| 64 | NV-2 | 38.93 | -114.41 | SH |  | nevcanspg1a |  |
| 65 | NV-3 | 38.90 | -114.33 | EN |  | nevcanspg2a nevcanspg4a |  |
| 66 | CA-1 | 38.41 | -120.95 | DB, GR | T10SFH | tonzi vaira |  |
| 67 | CA-2 | 38.11 | -121.54 | AG |  | bouldinalfalfa bouldincorn |  |
| 68 | CA-3 | 37.01 | -119.01 | EN | T11SLA | NEON.D17.TEAK.DP1.00033 | NEON.D17.SOAP.DP1.00033 |
| 69 | AZ-1 | 36.61 | -112.41 | EN, GR | T12SUF | segawhitepockets segalittlemountain | segasoapcreek grca1pj |
| 70 | AZ-2 | 35.59 | -111.97 | SH | T12SVE | segabluechute | flagstaffchimneysprings segabradshaw grca1pj |
| 71 | AZ-3 | 35.68 | -111.48 | GR |  | segablackpoint |  |
| 72 | AZ-4 | 35.72 | -111.35 | SH |  | segalcr segalcr2 |  |
| 73 | AZ-5 | 35.16 | -111.73 | GR, EN |  | segaarboretummeadow |  |
| 74 | AZ-6 | 31.74 | -109.94 | SH | T12SWA | kendall |  |
| 75 | AZ-7 | 31.82 | -110.87 | SH |  | srm |  |
| 76 | AZ-8 | 31.74 | -110.05 | SH |  | luckyhills |  |
| 77 | AZ-9 | 31.91 | -110.84 | SH |  | NEON.D14.SRER.DP1.00033 |  |
| 78 | AK-1 | 63.88 | -145.75 | EN, GR | T06VWR | NEON.D19.DEJU.DP1.00033 | NEON.D19.HEAL.DP1.00033 NEON.D19.BONA.DP1.00033 |
